# CAFs vs. TECs: when blood feuds fuel cancer progression, dissemination and therapeutic resistance

**DOI:** 10.1007/s13402-024-00931-z

**Published:** 2024-03-07

**Authors:** Diane Coursier, Fernando Calvo

**Affiliations:** Instituto de Biomedicina y Biotecnología de Cantabria (Consejo Superior de Investigaciones Científicas, Universidad de Cantabria), Santander, Spain

**Keywords:** Cancer, Tumor microenvironment, Endothelia, CAFs, Signaling, Crosstalk

## Abstract

Neoplastic progression involves complex interactions between cancer cells and the surrounding stromal milieu, fostering microenvironments that crucially drive tumor progression and dissemination. Of these stromal constituents, cancer-associated fibroblasts (CAFs) emerge as predominant inhabitants within the tumor microenvironment (TME), actively shaping multiple facets of tumorigenesis, including cancer cell proliferation, invasiveness, and immune evasion. Notably, CAFs also orchestrate the production of pro-angiogenic factors, fueling neovascularization to sustain the metabolic demands of proliferating cancer cells. Moreover, CAFs may also directly or indirectly affect endothelial cell behavior and vascular architecture, which may impact in tumor progression and responses to anti-cancer interventions. Conversely, tumor endothelial cells (TECs) exhibit a corrupted state that has been shown to affect cancer cell growth and inflammation. Both CAFs and TECs are emerging as pivotal regulators of the TME, engaging in multifaceted biological processes that significantly impact cancer progression, dissemination, and therapeutic responses. Yet, the intricate interplay between these stromal components and the orchestrated functions of each cell type remains incompletely elucidated. In this review, we summarize the current understanding of the dynamic interrelationships between CAFs and TECs, discussing the challenges and prospects for leveraging their interactions towards therapeutic advancements in cancer.

## Introduction

Neoplasia, a gradual process characterized by the accrual of genetic alterations enabling aberrant cell growth and survival, often requires additional cues for the transition to malignancy. These cues foster an environment conducive to cancer cell proliferation and acquisition of aggressive traits [1]. Tumors constitute intricate ecosystems comprising not only malignant cells but also non-malignant stromal components, such as endothelial cells (ECs), fibroblasts, and immune cells, alongside the extracellular matrix (ECM) and diverse soluble factors collectively referred to as the tumor microenvironment (TME) [2]. Significantly, the TME undergoes dynamic alterations in response to tumor growth, leading to notable changes in its physical, chemical, and cellular properties, thus instigating the acquisition of pathological behaviors. Consequently, stromal cells within the TME actively contribute to tumor initiation, malignant progression and metastasis [3]. Moreover, the TME profoundly influences therapeutic responses, encompassing both intrinsic and acquired reactions of the tumor stroma to therapies [2]. Recent insights highlight the intricate interplay between diverse TME constituents and their specific impacts on tumoral processes. Cancer cells adeptly modulate signaling and metabolic pathways to influence adjacent stromal cells, while each TME component (EC, fibroblast, immune cell, other specialized cell type) can reciprocally impact malignant cells and each other. Importantly, these interactions may vary depending on the tumoral process under evaluation, evolve during tumor progression, or respond differentially to distinct therapeutic interventions. Deciphering these networks holds promise in identifying pivotal nodes for targeted interventions and, at least in theory, the development of therapeutic approaches more likely to succeed. However, the complexity of these bidirectional interactions and their dynamic nature makes particularly difficult to obtain a holistic view of the network architecture to enable subsequent target identification.

Central to these intricate cellular interplays are cancer-associated fibroblasts (CAFs). These are highly abundant stromal cells capable of influencing both cancer and stromal cell behavior, as well as to significantly alter the physical-chemical properties of the TME [4–6]. Consequently, CAFs play integral roles in almost all cancer hallmarks, including cancer cell growth, survival, and invasiveness, thus exerting direct impacts on tumor development, dissemination, and therapy resistance [1, 4].

Here, we focus on delineating a specific facet of the complex TME network concerning the interactions between CAFs and the tumor vasculature. Tumor angiogenesis, a pivotal process in tumor progression, involves cancer cells hijacking diverse mechanisms to promote new blood vessel formation, sustaining tumor nourishment and oxygenation [7]. Notably, CAFs are known to secrete pro-angiogenic factors, stimulating this process and influencing EC behavior directly or indirectly. These modulations can alter the overall vascular architecture within tumors, potentially influencing metastasis and the efficacy of anti-cancer drug delivery. Conversely, TECs, despite presenting altered states influencing cancer cell growth and inflammation, remain relatively uncharacterized regarding their potential influence on CAF behavior.

This review summarizes the current knowledge on CAF-TEC interactions, delineating the ambivalent roles of CAFs in tumor vasculature and exploring the potential of TECs in modulating CAF phenotypes. Additionally, it examines how these interactions influence the efficacy of existing anticancer treatments, while outlining the challenges and opportunities to harness these insights for novel therapeutic interventions.

## Cancer-associated fibroblasts (CAFs)

### Description and origin of CAFs

Fibroblasts are traditionally defined as mesenchymal interstitial cells devoid of epithelial, endothelial or immune markers [4]. In general, fibroblasts are critical in maintaining tissue structure by generating and organizing connective tissue and orchestrating chemical and mechanical cues among diverse cellular components of the organ. In response to damage, quiescent fibroblasts assume an activated state pivotal for wound healing and tissue repair. Remarkably, this activation paradigm extends to neoplastic contexts, where the presence of activated fibroblasts, known as CAFs, represents a hallmark feature in numerous solid tumors. In parallel to the fibroblast definition, CAFs are cells of a mesenchymal lineage (non-epithelial, non-cancerous, non-endothelial and non-immune cells) that are located within or adjacent to a tumor [4, 6]. Importantly, CAFs are the most prominent stromal cell type in many solid tumors and have been shown to participate in tumor initiation, progression and metastasis, and their content within tumors is associated with worse prognosis.

**Fig. 1:**
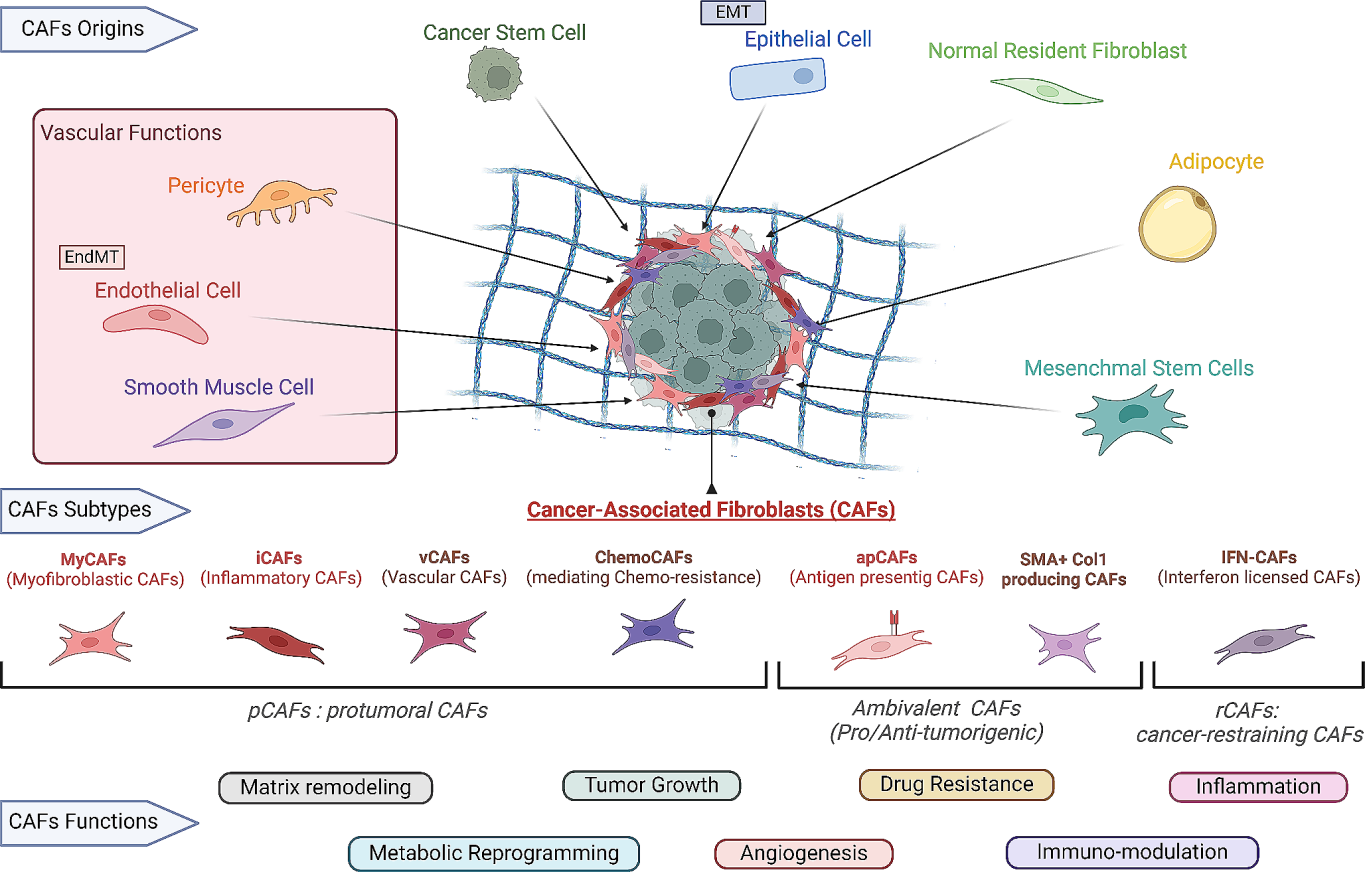
Origins of CAFs and CAF functions within the TME. Diagram showing the different stromal cells that can be hijacked and transformed into CAF, and the different CAFs subtypes implicated in tumor progression through a wide range of pro-tumor functions

The broad definition of CAFs encompasses their diverse cellular origin and likely impacts in their high level of heterogeneity (Fig. 1). CAFs have been shown to originate primarily from resident tissue fibroblasts (or stellate cells) or from mesenchymal cells recruited to the TME from the bone marrow. Alternatively, CAFs may also originate from alternative resident cells including mesenchymal stem cells, adipocyte-derived precursor cells, ECs, mesothelial cells or pericytes [8]. Nonetheless, unequivocally defining the precise origin of CAFs remains elusive due to their inherent plasticity and the lack of well-defined lineage biomarkers. Regardless of their cellular origin, CAFs present a notorious altered state when compared to their normal precursors.

CAFs are generally regarded as genetically stable cells and their altered state is thought to be a cellular response to the myriad of stresses present in the TME. These insults include physiological stress, changes in the composition of chemokines/cytokines (i.e. inflammatory signals such as TGFβ, IL1, IL6, TNFα), presence of other soluble ligands (PDGF, FGF, Wnt), ECM composition and mechanical properties, or cell-cell interactions [6]. When applied to a CAF precursor, these insults promote phenotypic changes associated with CAF emergence or activation. As a result, there is an expanding and diverse list of molecular mechanisms associated with the regulation of CAF phenotypes [9]. Notably, these mechanisms encompass pivotal regulators such as SMAD transcription factors, inflammatory signaling pathways (e.g., JAK/STAT, NF-κB), mechanotransduction cascades (e.g., YAP/TAZ, SRF), stress-responsive pathways (e.g., HSF1, ATF4), Notch signaling, as well as canonical pathways (e.g., ERK, Akt, Wnt/β-catenin). Ongoing research is also uncovering additional cellular processes influencing CAF emergence, including metabolic reprogramming (e.g., autophagy, glycolysis) [10], activation of embryonic programs (e.g. Twist) or interaction with immune-modulators such as IFNγ [11]. Importantly, the pathological activation of CAFs suggests the establishment of intricate feed-forward signaling circuits and/or epigenetic alterations perpetuating their aberrant state.

### Impact of CAFs in tumor development

The altered phenotype of CAFs is coupled with significant changes in their cellular behavior and functions, exerting direct and indirect influences on multiple pro-tumoral processes [4, 5] (Fig. 1). Consequently, through ECM remodeling and signaling interactions with cancer, endothelial, and immune cells, CAFs emerge as pivotal orchestrators shaping the physical and chemical TMEs [5, 6].

Analogous to fibrotic disorders, the pathological activation of fibroblasts in tumors correlates with excessive ECM deposition and remodeling. Within the tumor ECM, CAFs play a central role in generating a dense network of collagen fibers, hyaluronan, glycosaminoglycans, and proteoglycans, substantially altering its composition [12]. Additionally, CAFs secrete matrix crosslinkers like LOX and matrix metalloproteases (MMPs), further contributing to ECM remodeling. This desmoplastic ECM functions as a signaling platform, influencing cancer cell behavior by instigating pro-survival and proliferative signaling pathways. Notably, compared to quiescent fibroblasts, CAFs exhibit cytoskeletal rearrangements resulting in increased cellular contractility, generation of mechanical forces and migration [13, 14]. The combined action of ECM production/remodeling, and the generation of physical forces by CAFs engenders ECM reorganization that significantly influences tumor behavior. This includes orienting fibers to promote cancer cell motility, creating permissive tracks facilitating cancer cell invasion, and augmenting tissue stiffness, promoting malignant phenotypes in cancer cells [15–17]. Importantly, these ECM alterations extend their impact to other TME stromal cells, hindering leukocyte infiltration and influencing tumor vasculature [18].

Moreover, CAFs serve as crucial signaling hubs, secreting an array of growth factors, cytokines, and exosomes that modulate tumor behavior [19]. This diverse secretome fosters proliferative and invasive behaviors in cancer cells, impacts their self-renewal capacities and enhances pro-survival signaling, establishing a link between CAF action and tumor progression, dissemination and therapeutic responses in tumors [20]. Additionally, CAF-derived factors profoundly affect various TME components, specifically impacting EC behavior (*see* Sect. 4). Furthermore, CAF-secreted cytokines and chemokines can act on an expanding list of leukocytes and modulate inflammation and immunosuppression, particularly impacting T-cell recruitment and activity, and influencing outcomes of immunotherapies [11]. In agreement, Krishnamurty et al. recently illustrated how targeting a specific population of CAFs could be an attractive therapeutic strategy for improving immunotherapy outcomes. Briefly, the study demonstrated that TGFβ signaling promotes the emergence of a CAF subpopulation characterized by Lrrc15 expression. Depletion of this CAF subset by genetic means resulted in a significant improvement in the intratumoral CD8^+^ T cells infiltration and effector function in pre-clinical pancreatic ductal adenocarcinoma (PDAC) models, and potentiated the efficacy of anti-PDL1-based therapeutics [21].

Furthermore, CAFs exhibit altered expression of cell surface proteins and receptors fostering new cell-cell interactions. These interactions, when established with cancer cells, contribute to local invasion [22, 23]. Additionally, CAFs express molecules associated with antigen presentation, influencing T-cell activation/suppression [24, 25]. CAFs may also alter the TME through metabolite and amino acid exchanges. Thus, metabolites produced by CAFs fuel cancer cell growth upon uptake, while changes in metabolite composition influence immune cell behavior [10].

Noteworthy, original studies employing targeted elimination of CAFs in preclinical models already hinted at the existence of CAFs (or particular CAF subsets) with tumor restraining activities. Thus, genetic elimination of Fap^+^ fibroblasts was associated with enhanced anti-cancer immunity and slower tumor growth in preclinical models of lung cancer [26], similar to phenotypes associated with Lrcc15^+^ CAFs. On the other hand, destruction of αSMA^+^ CAFs showed opposite results with the generation of undifferentiated, immune suppressed, highly aggressive tumors [27]. More recently, the particular tumor restraining phenotypes in αSMA^+^ CAFs have been better defined and associated to type I collagen production by this subpopulation. Thus, *Col1* deletion in αSMA^+^ CAF in PDAC models induces immune suppressive signaling in neighboring cancer cells [28]. Furthermore, using different experimental liver metastasis models, Bhattacharjee, et al. proposed that CAF-expressed type I collagen could suppress tumor growth by mechanically restraining tumor spread [29]. Noteworthy, contradictory results have been observed in regards to the tumor suppressive role of SMA^+^ and Col1-producing CAFs in other models [30, 31]), underlying the complexity of the system. Finally, a distinct subset of MHCII-expressing, antigen-presenting CAFs have shown to directly promote MHCII immunity through effector CD4 T cell activation, showing tumor-suppressive immune potentiating properties [24].

### CAF heterogeneity and subpopulations

The diversity in cellular origins and regulatory influences on CAF behavior suggests inherent heterogeneity and plasticity among these cells. Additionally, ascribed functions to CAFs hint at nuanced specialization among these cells, a phenomenon gaining attention in recent investigations [8]. Studies leveraging single-cell RNA sequencing (scRNAseq) techniques on cell populations from animal models and clinical samples have begun unveiling distinct CAF subtypes based on their gene expression profiles. The identification, characteristics, and composition of these subpopulations vary across models, tumor types, and stages, highlighting the plethora of factors influencing CAF behavior. Despite these variances, a consensus has emerged, leading to the classification of CAFs into different subgroups (Fig. 1) [6, 8].

Firstly, myofibroblastic CAFs (myCAFs) represent a CAF subset enriched in ECM production and remodeling with contractile properties and heightened TGFβ signaling. Within this group, some studies classified ECM remodeling, wound healing-associated features (e.g. contractility) and TGFβ-signaling as distinct subgroups [32]. Typically associated with pro-tumoral functions, myCAFs often correlate with poor prognosis. However, myCAFs may contain additional phenotypes and functions, as exemplified by the paradoxical effects of their targeted elimination in preclinical models explained before. Thus, certain myCAFs and TGFβ-responsive CAFs have been implicated in immune suppression [21, 32, 33], whereas Col1-expressing myCAFs have shown to present tumor restrictive properties [28, 29].

Secondly, the inflammatory/immune regulatory CAFs (iCAFs) constitute a CAF population displaying elevated levels of chemokines/cytokines and activation of inflammatory pathways like NF-κB. These fibroblasts secrete a spectrum of inflammatory, immune-modulatory, and chemoattractant mediators along with elements linked to complement regulation. The diverse array of immune-modulatory functions observed in iCAFs suggests additional levels of specialization that still require further investigation, but in general it has been shown that they generate signals that promote cancer cell growth, inflammation and immune suppression.

The third subgroup comprises antigen-presenting CAFs (apCAFs), marked by their characteristic expression of antigen presentation-related molecules, notably MHC-II. Initially associated with the atypical antigen-presenting cell group, apCAFs may participate in T-cell stimulation. However, their role in providing requisite signals for complete activation of naïve T cells or potential interference with immune responses remains unclear [25, 34].

Additionally, there exist “rare” CAF subpopulations observed in specific tissues, models, or arising due to particular interventions. For instance, some studies have described the presence of CAFs in the vicinity of blood vessels in tumors with expression of certain vascular genes, the vascular CAFs (vCAFs) [35, 36]. Another newly characterized CAF subtype, termed “chemoCAFs,” expresses abundant chemokines but lacks the typical inflammatory cytokine profile associated with iCAFs (i.e. IL1, IL6, IL11, LIF or VEGFA), indicating a distinct inflammatory role [37]. Notably, TGFβ blockade in mouse tumors resulted in the emergence of a fibroblast population termed “interferon-licensed CAFs”, exhibiting robust interferon response and increased immune-modulatory properties [38].

## Key features of tumor vasculature and its impact in tumorigenesis

### Regulation of blood vessel formation

Blood vessels play a pivotal role in tissue physiology by facilitating the transport of essential nutrients, oxygen, metabolites, and cells throughout the organism to uphold homeostasis [39]. In embryonic development, nascent vessels arise through the differentiation of EC precursors, culminating in the formation of a primitive vascular network (i.e. vasculogenesis). Subsequent expansion of this network occurs via vessel sprouting and remodeling, orchestrated by a coordinated network of signals that stimulate EC proliferation and angiogenesis. These specialized ECs form the inner lining of vessels, enwrapped by a specialized basement membrane and surrounded by vascular mural cells (comprising smooth muscle cells, pericytes, and fibroblasts), ensuring vascular stability and required perfusion for optimal blood flow and pressure. Notably, these vascular supporting cells as well as the vascular ECM are crucial for the formation of functional vascular networks [40, 41]. This process is mediated by various mechanisms, including paracrine signaling, specialized ECM generation, direct cellular contact and contractility, and modulation of tissue physical properties [41, 42].

In the healthy adult, vasculature remains largely quiescent, exhibiting minimal branching to maintain an effective barrier against liquid and cell extravasation. However, ECs retain a remarkable degree of plasticity and responsiveness to external signals that can modulate their behavior. In response to inflammatory cues, ECs enhance immune cell trafficking during infection or tissue injury. Alternatively, prompted by pro-angiogenic signals, ECs exhibit proliferative and motile behaviors, giving rise to new sprouts that evolve into fully functional vessels [39]. These processes are integral during physiological events such as development or wound healing and are tightly regulated, promptly ceasing once the initiating stimulus diminishes or the requisite vasculature formation is achieved. Nevertheless, dysregulation of these mechanisms actively contributes to various pathological conditions, including inflammatory diseases and cancer.

### Tumor angiogenesis

The formation of new vessels represents a critical event associated with the rapid expansion of tumors, both at primary sites and in metastases. Neovascularization plays a crucial role in sustaining tumor growth beyond 1–2 mm in diameter, overcoming limitations in nutrient and oxygen diffusion to the central regions of the tumor [39]. Consequently, tumors have evolved strategies to foster angiogenesis, ensuring proper nutrient and metabolite transport. Tumor neovascularization is initiated by an event termed the “angiogenic switch”, triggered either by direct actions of cancer cells or alterations within the chemical composition of the TME, such as hypoxia. This switch instigates the production of proangiogenic molecules, notably vascular endothelial growth factor (VEGF)-related factors within the TME. These factors bind to their corresponding VEGF receptors expressed on ECs, directly stimulating EC proliferation, migration, and angiogenesis [43]. Additionally, several other pro-angiogenic factors contribute to EC proliferation and migration. For instance, platelet-derived growth factors (PDGFs) recruit stromal fibroblasts that secrete VEGF, thus fostering angiogenesis [44], which is a mechanism shared by other factors such as TGFβ. Moreover, PDGFs recruit progenitor ECs, induce their maturation, and promote pericyte migration to vessel walls, all contributing to tumor angiogenesis. Intriguingly, the impact of PDGFs on ECs may be reliant on other pro-angiogenic factors like the fibroblast growth factor (FGF) family. FGF2 stimulation in ECs promotes the expression of PDGFα and -β receptors, fostering a synergy between both signaling pathways [45]. Furthermore, FGFs, upon binding to their receptors on ECs, directly instigate angiogenesis in vitro and in vivo [46]. Hepatocyte growth factor (HGF) is another pro-angiogenic factor contributing to the angiogenic phenotype in ECs, particularly in early angiogenic steps, including cell migration and proliferation [47].

Additionally, juxtacrine signaling contributes to aberrant tumor angiogenesis. Notch signaling emerges as a critical regulator in angiogenesis, participating in both the initiation and cessation of angiogenesis through distinct mechanisms. Notably, Notch signaling promotes EC differentiation by stimulating tip cell specification and plays a pivotal role in EC behavior modulation. In cancer, overexpression of the Notch ligand Jag1 in cancer cells activates Notch signaling in TECs, enhancing angiogenesis through proliferation and vessel stabilization. Conversely, the Notch ligand DLL4, primarily expressed by TECs, potentiates their proliferation and migration [48, 49].

### TEC characteristics

The pathological imbalance in EC signaling pathways induces perturbations in TECs, significantly impacting their function and influencing tumor progression. Transcriptional signatures of TECs exhibit variations based on anatomic location, tumor type, and stage. However, they commonly exhibit upregulated genes associated with developmental and physiological angiogenesis [50]. TECs often manifest pro-angiogenic behaviors characterized by increased motility, proliferation, and concomitant EC anergy (*see below*). In addition, TECs may develop proinflammatory states characterized by the upregulation of adhesion molecules and cytokines/chemokines. TECs also have irregular morphologies with reduced cell-cell junctions which affect their barrier function [51], metabolic rearrangements (e.g. higher RNA content, increased aerobic glycolysis) [52]; as well as chromosomal abnormalities, including aneuploidy, deletions and translocations. Some of these phenotypes may contribute to an increased self-renewal capacity, partially fueling neovasculogenesis. The array of alterations contributes to marked heterogeneity among ECs in terms of signaling and gene expression profiles, a landscape currently being unveiled through scRNAseq profiling [50].

### Tumor vessel structural abnormalities

**Fig. 2:**
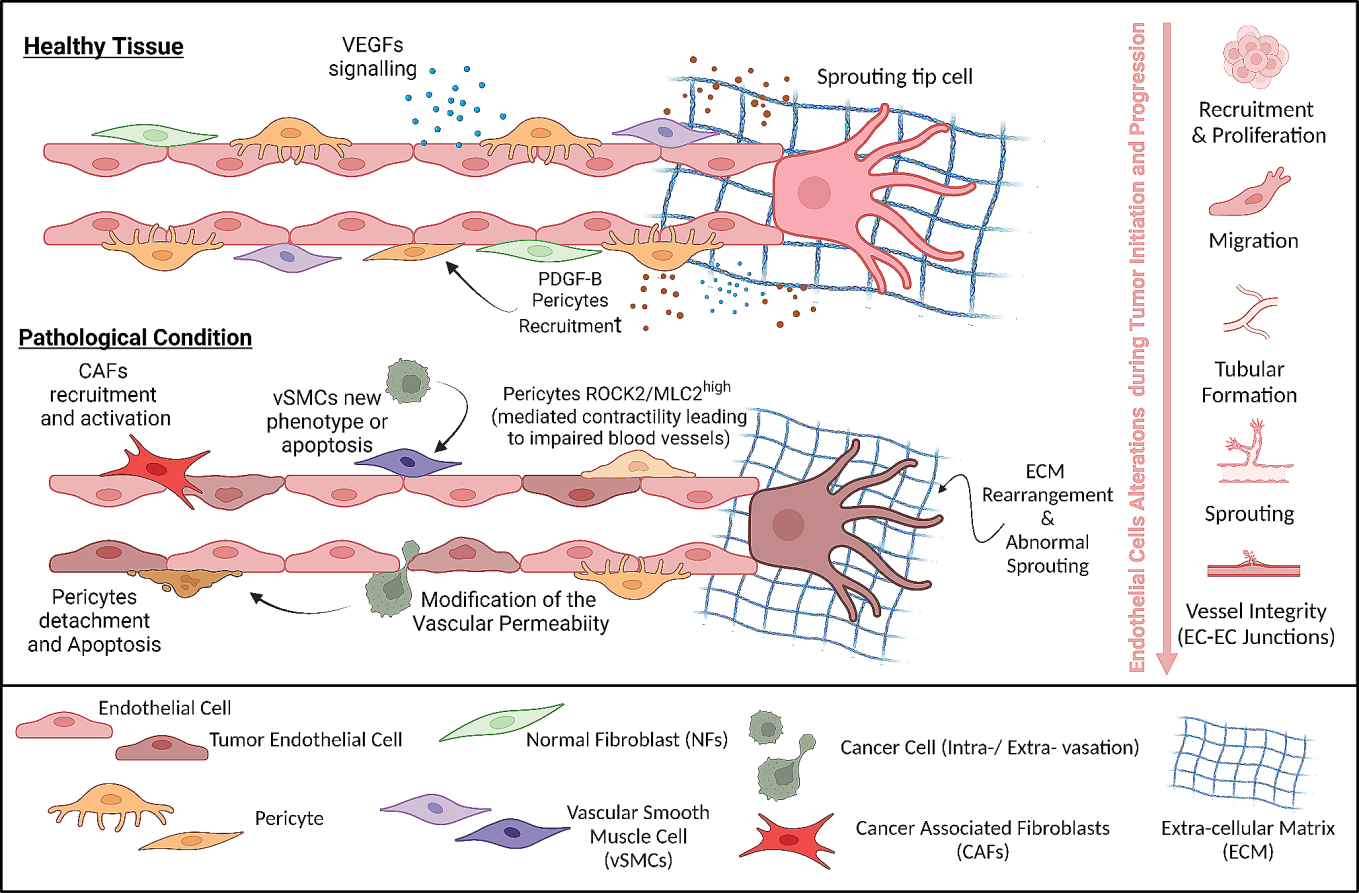
Tumor vessel abnormalities. Schematic representation of the main features of blood vessels in normal and pathological (i.e. cancer) conditions. In healthy tissues, the integrity of the vessels is maintained by the presence of the perivascular stromal cells. In cancer, the TME is inducing changes in mural cell phenotypes through apoptosis, detachment or activation, as well as recruitment of CAFs. In addition, alteration in TEC characteristics results in alterations in vascular integrity increasing vessel leakiness and allowing cancer cells to intra- and extravasate

Tumor vessels exhibit an excessively branched and disorganized architecture due to abnormalities in TECs, leading to an unstable vessel wall that promotes vascular leakage and reduced perfusion [53]. In addition, this chaotic organization of blood vessels limits effective blood distribution throughout the tumor parenchyma, resulting in areas of persistent or intermittent hypoxia (Fig. 2).

Vascular integrity in tumor vessels is frequently compromised, as evidenced by disrupted endothelial junctions, causing heightened permeability and accumulation of interstitial fluid pressure [54]. Mechanistically, Vascular Endothelial-cadherin (VE-cadherin), a critical endothelial adhesion molecule, is located at EC junctions to regulate cell-cell contacts. During inflammatory processes, phosphorylation modifications to VE-cadherin disrupt interconnections between adjacent ECs, facilitating alterations in leukocyte infiltration [55]. In agreement, stimulation of artificial EC networks with the pro-inflammatory factor TNFα results in increased EC permeability, enhancing tumor cell transendothelial migration [56]. Furthermore, altered TEC behavior and vascular architecture may result from substantial changes in the vascular ECM lining the vessels. The ECM orchestrates complex signaling cascades within ECs, influencing pivotal aspects of their biology, including proliferation, migration, cell shape, survival, and ultimately, blood vessel stabilization [57]. Additionally, the ECM stores factors with both pro- and anti-angiogenic activity, requiring proteolytic processing to become active. Excessive ECM deposition and remodeling associated with tumor desmoplasia alter these regulatory mechanisms, promoting EC angiogenesis and inflammation [18]. Desmoplastic ECM may exert mechanical forces on blood vessels, affecting their integrity and leading to altered vascular function.

Moreover, defects in mural cell coverage profoundly affect vascular integrity. Prevention of pericyte recruitment by targeting PDGFβ signaling alters vascular architecture in tumors and increases metastatic dissemination [58]. A tight regulation of the contractile capacity of the pericytes is also essential for vessel functioning and integrity [59], and abnormal vascular networks in cancer may also emerge from alterations in the behavior of mural cells rather than their absence. In support of this notion, it has been recently described that, compared to normal tissue pericytes, tumor-associated pericytes present elevated ROCK2-MLC2 mediated contractility leading to impaired blood vessel supporting function and decreased drug perfusion [60]. On the other hand, inhibition of Notch signaling in mural cells induces a downregulation of their contractile activity and the establishment of a secretory phenotype associated with abnormal vasculature [61].

### Other roles of TECs in tumorigenesis

Importantly, TECs may also influence tumor progression independently of their fundamental vascular function. The irregular and permeable architecture of tumor vessels enables the intravasation and metastatic spread of cancer cells as their barrier function is diminished through the downregulation of intercellular adhesion molecules like VE-cadherin and PECAM, alongside inadequate pericyte coverage [58, 62]. TECs utilize MMPs to degrade vessel basement membranes, facilitating the infiltration of migratory cancer cells into the vasculature [63]. Additionally, elevated adhesion molecule expression by TECs serves as an anchor for metastatic cancer cells, promoting intravasation and metastasis [64]. TECs also influence cancer growth and dissemination by secreting factors (e.g., IL3/6/8, FGF, PDGF, TGFβ) that directly trigger cancer cell proliferation and migration [51]. These interactions can significantly impact responses to therapeutic treatments; for instance, chemo-resistant disseminated tumor cells often occupy perivascular niches at distant sites, evading therapy by interacting through integrins with VCAM-1 on the microvascular endothelium, enabling survival independently of cell-cycle status [65].

Moreover, TECs emerge as key regulators of immune responses in tumors, akin to their pivotal role in immune surveillance under normal conditions [66]. Thus, abnormal leaky tumor vasculature represents an obvious hurdle for immune surveillance cells to reach certain tumoral areas [67]. In addition, TECs also play an active role in modulating immune cell adhesion and infiltration, as well as participating in processes affecting immune cell priming and activation, thereby directly impacting anti-cancer immune responses [51]. Under normal conditions, quiescent ECs do not signal for leukocyte recruitment; however, upon stimulation, they express adhesion molecules (e.g., E/P-selectin, VCAM1/ICAM1) and release chemokines (e.g., CCL2, CCL18, CXCL10, and CXCL11) facilitating leukocyte arrest and transmigration into tissues. These activations typically involve inflammatory signals like TNFα, IFNγ, and interleukins, predominantly via NF-κB, STAT1/3, and STING pathways [51]. TGF-β signaling induces vascular inflammation and permeability by upregulating proinflammatory chemokines, cytokines, adhesion molecules, and fibronectin [68]. Intriguingly, TECs show reduced sensitivity to these immune stimulatory mechanisms through “endothelial anergy” [69]. Induced by various tumor-secreted cytokines and chemokines, particularly pro-angiogenic factors like VEGF family members and FGF2, this phenotype hinders responsiveness to pro-inflammatory signals, actively downregulating pro-immune adhesion molecules and chemokines. Hence, endothelial anergy and tumor angiogenesis primarily associate with TECs, potentially linking aberrant angiogenesis with EC-mediated immunosuppression.

Furthermore, TECs may contribute to cancer-related immune suppression by directly affecting T cell activation through inhibitory mechanisms. They express immune checkpoint molecules (e.g., PD-L1, PD-L2, TIM3), alongside other molecules (e.g., FasL, IDO1) inhibiting T cell function in response to pro-inflammatory cytokines [51]. Additionally, a potential role of ECs as non-professional antigen presenting cells is emerging, as certain subtypes express genes associated with MHC-II-mediated antigen presentation. Interestingly, it appears that TECs can downregulate these genes, suggesting an additional mechanism for immune suppression [70]. Finally, certain EC subpopulations can also participate in the formation of tertiary lymphoid structures, which have been shown to affect anti-tumor immunity responses in certain tumor types [71].

However, it is crucial to recognize that ECs within tumors can adopt inflamed phenotypes, contributing to tissue inflammation and cancer aggressiveness by: (i) producing proinflammatory factors such as IL6 that induce macrophage polarization [72]; (ii) reduced expression of VE-cadherin and CD31 alongside increased expression of adhesion molecules, that facilitate leukocyte extravasation and metastasis [73]. Accordingly, inflamed EC markers such as ICAM-1 and VCAM-1 have been correlated with cancer aggressiveness [73]. In addition, sustained endothelial Notch1 signaling generates a senescent, pro-inflammatory endothelium in tumors and promotes metastasis [48]. Intriguingly, inflamed ECs are not proliferative and therefore will oppose pro-angiogenic behaviors (and vice versa). This aspect should be considered when interpreting in vitro EC models (e.g., Human Umbilical Vein ECs– HUVECs–, Human dermal Microvascular ECs– HMVECs) predominantly maintained in pro-angiogenic culture conditions to facilitate sufficient proliferation for assay preparation. Moreover, as the TME encompasses both pro-angiogenic and pro-inflammatory signals, these observations suggest that angiogenic and inflamed TECs may coexist in different areas of the TME, or predominate at various tumor development stages. Supporting this, a study reported that inflamed ECs inhibited primary tumor growth while promoting metastasis [74].

Overall, an expanding body of evidence suggests that TECs may have unexpected roles in tumor progression and dissemination, alongside emerging involvement in immune suppression, potentially interfering with anti-cancer therapies. Analogous to CAFs, technological advances enabling the isolation and characterization of TECs, as well as the identification of different TEC subpopulations with specialized functions, are imperative. These will significantly improve our understanding of TECs, allowing assessment of their potential as prognostic/predictive factors, and identifying appropriate clinical/therapeutic settings and strategies for TEC modulation.

## Role of CAFs in modulating tumor vasculature

The capacity of tumors to promote neo-angiogenesis to fuel their growth is not solely attributed to malignant cells; other constituents within the TME, particularly CAFs, play a significant role in this process due to their structural and signaling importance within the TME, which can significantly affect the process through different mechanisms. Nevertheless, discerning the overall impact of CAFs on tumor angiogenesis remains challenging, as they exhibit both positive and detrimental effects on angiogenesis, tumor vascularization, and blood perfusion.

### Angiogenic functions of CAFs: production of pro-angiogenic factors

Seminal research by Orimo et al. highlighted the capacity of CAFs to drive tumor angiogenesis through increased production of SDF-1/CXCL12 compared to normal fibroblasts, thereby promoting recruitment and activation of progenitor ECs within the TME [75]. Subsequent studies have reinforced the pivotal role of CAFs in tumor angiogenesis by secreting various pro-angiogenic factors [4, 5]. Foremost among these is the ability of most CAFs to serve as the primary source of VEGF-related factors within tumors. Additionally, CAFs demonstrate the capability to produce other mediators of tumor angiogenesis, including members of the PDGF and FGF families, HGF, and various interleukins. Some of these factors might act in an autocrine manner to enhance VEGF expression in CAFs or initiate alternative pro-angiogenic signaling cascades. For instance, PDGF receptor signaling in CAFs can induce the expression of FGF7 and FGF2, directly contributing to tumor angiogenesis, as evidenced by the impairment of angiogenic phenotypes when FGF ligand traps were used [76].

Furthermore, specific factors secreted by CAFs have been implicated in promoting tumor angiogenesis, although it remains unclear whether they directly influence EC behavior or foster pro-angiogenic factor production. For example, Unterleuthner et al. demonstrated that colon CAFs exhibit upregulated expression of WNT2, a factor involved in placental vascularization. Notably, WNT2^high^ CAFs stimulated EC migration, fostering colon cancer progression by inducing the WNT2-dependent upregulation of pro-angiogenic factors such as IL6, G-CSF, and PGF [77]. In line with these observations, a recent study further implicated Wnt signaling regulation in CAFs with the modulation of angiogenesis in tumors [78]. The authors identified a paracrine cascade involving Sonic-Hedgehog (SHH), Wnt and VEGF signals that limits angiogenesis in PDAC. Briefly, KRAS activation in PDAC leads to SHH production by cancer cells and paracrine activation of GLI transcription factors in CAFs, which induce the expression and secretion of the Wnt antagonist WIF. As a result, VEGF production downstream of Wnt signaling is restrained, thereby limiting VEGFR2-dependent activation of endothelial hyper-sprouting. On the other hand, SHH signaling inhibition releases the pro-angiogenic activity of WNTs and leads to VEGFR2 activation in ECs. Other factors produced by CAFs may also induce angiogenesis. CAF-secreted IL11 and IL15 have been shown to activate STAT3 signaling in ECs, promoting pro-angiogenic behaviors independently of VEGF [79]. Additionally, chemokines like CXCL8 and CCL2 produced by CAFs have been implicated in inducing EC tube formation in vitro [80].

### Alternative pro-angiogenic functions of CAFs

CAFs may not only induce angiogenesis by producing angiogenic factors, but also via ECM generation and remodeling. CAFs play a pivotal role in producing crucial matrisome components necessary for vascular formation [81]. These components encompass various molecules like TNC, known to prompt pro-angiogenic signaling [82]; CYR61, which facilitates adhesion, migration, and proliferation of ECs [83]; and OPN, that induces angiogenesis through activation of PI3K and ERK in ECs [84]. Additionally, CAFs exhibit increased expression of multiple MMPs capable of degrading ECM, releasing stored soluble factors, and thereby altering the signaling properties of the TME and affecting angiogenesis. This has been documented for MMP13, which in a model of skin carcinoma was shown to be induced in fibroblasts and correlated with the expression of VEGF [85]. In tumors grown in *Mmp13* ^−/−^ mice, the authors showed reduced VEGF expression in the stroma as well as impaired tumor growth and vascularization. Similar functions are anticipated for other MMPs upregulated in CAFs such as MMP2 and MMP9.

Furthermore, CAFs may stimulate angiogenesis independently of secreted factor production. In in vitro models of vasculogenesis, breast CAFs were observed to enhance vascularization when compared to normal fibroblasts, partly by upregulating VEGF and also through their ability to generate forces, producing substantial deformations in 3-dimensional gels [86]. Inhibition of force generation reduced CAF-induced matrix deformation and suppressed vascularization, while artificial induction of ECM deformations partially restored it. Mechanotransduction pathways in CAFs, possibly activated due to changes in ECM mechanical properties or cytoskeletal remodeling, might also induce abnormal angiogenesis by triggering the production of pro-angiogenic factors. For instance, YAP activation in CAFs, in response to changes in ECM mechanics or cytoskeletal alterations, can foster in vivo angiogenesis by upregulating VEGFA, VEGFC, and TGFβ1 [13, 14]. Similarly, Du et al. reported that YAP activation in fibroblasts could promote VEGF-independent angiogenesis via IL11 and IL15 expression, enhancing tube formation even during anti-angiogenic treatment (AAT) with Axitinib [79].

While CAFs predominantly express pro-angiogenic factors, they may also express anti-angiogenic molecules, albeit the evidence in this aspect is relatively limited. For instance, CAFs have shown an upregulation of Thrombospondin-1 and − 2 (THBS1/2) [14], glycoproteins implicated in cell-cell and cell-ECM interactions. THBS1/2 are known to negatively impact VEGF signaling, inhibiting EC migration, proliferation, and capillary tube formation [87, 88].

### Angiogenesis-independent functions of CAFs on ECs

The fundamental function of CAFs as key modulators of the physical-chemical properties of the ECM suggest additional roles in the modulation of tumor vasculature independent of their well-defined angiogenic functions. In particular, the exacerbated ECM deposition by CAFs and enhanced cellular contractility contribute to tissue stiffness and solid stress, which can compress vessels and affect tumor vessel architecture. In fact, it has been described that dense ECMs generate mechanical forces that squeeze blood vessels, compromising their integrity and hindering blood perfusion [18]. Moreover, CAFs undergo cytoskeletal rearrangements and possess substantial cellular contractility, impacting ECM remodeling and tissue stiffness independently of ECM deposition [14], which may directly affect vessel compression, akin to the effect observed with tumor-associated pericytes [60]. Additionally, CAFs modulate EC-EC junctions, enhancing EC motility and permeability, thereby increasing microvessel leakiness within the tumor [89].

Their characteristic enhanced contractility also endows CAFs with increased migratory potential, enabling them to move through interstitial tissue, potentially facilitating cancer cell invasion [16]. Through this process, CAFs may reach the vascular bed and digest its ECM. Given the critical role of the vascular basement membrane in regulating vessel permeability, resistance to compression, and vessel perfusion [90], CAFs may significantly impact the tumor vasculature through these mechanisms [57]. Importantly, these processes may also induce the detachment of the mural cells supporting the vessels, leading to weakened EC-EC junctions and increased vessel leakiness. Moreover, solid stress exerted by CAFs can compress lymphatic vessels, elevating interstitial fluid pressure and exacerbating the reduced blood flow caused by vessel leakiness. Indeed, strategies aiming at inactivating or eliminating CAFs have demonstrated vessel decompression and increased tumor vascularity [91, 92], aligning with similar findings observed in other contexts like vascular injury, where inhibiting TGFβ-mediated myofibroblast activation reduced vessel constriction [93] *(Discussed in more detail in* Sect. 7).

Certainly, it is crucial to acknowledge that these mechanisms might work concurrently with pro-angiogenic processes, illustrating the interconnectedness between heightened angiogenesis and abnormal vessel architecture in tumors. For instance, increased collagen content in tumors has been linked to angiogenesis and could be associated with ECM stiffness [94]. Moreover, CAF mechanotransduction and ECM remodeling have demonstrated the ability to enhance vessel formation both in vitro [86] and in vivo [13, 14], highlighting their role in promoting angiogenesis alongside structural changes in the tumor vasculature.

Apart from these relatively well-established roles, CAFs can also influence TECs by modulating tumor inflammation. Particularly, the inflammatory CAF (iCAF) subpopulation contributes to generating an inflammatory TME that might impact the behavior of TECs by promoting inflammatory states [8, 95]. CAFs, including iCAFs, have been observed to secrete factors directly implicated in EC inflammation, such as TNFα and interleukins [51]. This function might be further amplified indirectly through the role of CAFs in shaping the immune cell compartment towards inflammatory or immune-suppressive states via the secretion of cytokines, chemokines, and reactive oxygen species. Thus, studies in other contexts have established a critical role of fibroblasts in vascular inflammation and related pathologies [73, 96]. However, the specifics of this interaction in tumoral contexts remain unclear and require further investigation.

### Regulatory mechanisms of CAF interaction with TECs / CAFs specialization

It is evident that a common characteristic of CAFs lies in the upregulation of pro-angiogenic factors, fostering EC recruitment, migration, proliferation, and differentiation. Various dysregulated mechanisms in CAFs have been closely associated with the production of angiogenic factors. For instance, studies have demonstrated that TGFβ signaling in CAFs plays a pivotal role in inducing the secretion of VEGF [97]. Additionally, inflammatory signaling pathways like NF-κB stimulate the production of pro-angiogenic ECM factors such as CYR61 and OPN, consequently impacting vascularization in vivo [95]. As previously mentioned, other crucial signaling pathways in CAFs, such as PDGF or mechanotransduction pathways like YAP, can also induce the expression of pro-angiogenic factor [13, 14, 76]. Similar to ECs, hypoxia can also induce the expression of VEGF in CAFs via HIF1α/GPER [98]. Interestingly, less-characterized mechanisms underlying CAF functions may significantly impact their pro-angiogenic behavior. Stress responses mediated by ATF4 have recently been associated with the ability of CAFs to drive angiogenesis and tumor progression in melanoma and pancreatic cancer models [99]. along with reduced tumor growth and decreased expression of perivascular CAF activation markers (e.g., ACTA2, PDGFRβ, FAP). Notably, ATF4 deficiency in CAFs diminished the expression of pro-angiogenic factors, subsequently affecting their capability to promote tube formation in ECs. This underscores the multifaceted roles of CAFs and the diverse mechanisms through which they regulate angiogenesis in the TME.

In terms of CAF specialization, both myCAFs and iCAFs demonstrate angiogenic characteristics by actively producing pro-angiogenic factors. For instance, in addition to the known positive effect of myCAF inducer TGFβ on VEGF production, gastric αSMA-positive CAFs, a well-characterized myCAF marker, have been identified to produce Galectin-1. This Galectin-1 has been shown to stimulate EC proliferation, migration, and tube formation potential [100]. On the other hand, iCAFs contribute to angiogenesis by secreting a diverse array of chemokines, cytokines, and soluble factors that are anticipated to promote angiogenesis. For instance, cancer cells can induce the generation of iCAFs by releasing IL1β, which activates the LIF/JAK/STAT3 axis and subsequently triggers the expression of IL6 [101]. The iCAF population is characterized by its high production of IL8, an interleukin that also fosters angiogenesis and induces vessel permeability. Furthermore, in colon cancer, CAFs expressing IL6 have been identified to produce VEGF and instigate angiogenesis [102]. However, beyond their roles in directly promoting angiogenesis, myCAFs and iCAFs may impact tumor vasculature through distinct angiogenesis-independent mechanisms, as previously discussed. MyCAFs might be specifically involved in mechanisms related to ECM remodeling and vascular compression, whereas iCAFs could participate in processes linked to EC inflammation. This underscores the diverse ways in which these CAF subtypes contribute to the modulation of tumor vasculature.

Overall, studies have indicated that the potential to influence tumor vasculature is a general feature of CAFs, albeit with minor differences in the precise processes affected and the underlying mechanisms. However, there is still limited data available on the identification and characterization of specific pro- or anti-angiogenic CAF subpopulations. For instance, in colorectal cancer, KRAS-transformed cells generate pro-adipogenic factors (BMP4 and WNT5B) triggering the emergence of lipid-rich CAFs that produce VEGF and promote angiogenesis [103]. Regarding anti-angiogenic CAFs, an analysis of CAF populations in hepatocellular carcinoma identified a CAF subtype producing large amounts of Prolargin, which could bind and inhibit the activity of several pro-angiogenic factors (e.g. HGF and FGF) [104]. A recent significant finding is the identification of a novel CAF subtype with vascular features called vascular CAFs (vCAFs) [35]. Intriguingly, these cells did not express EC markers but presented increased expression of vascular regulators such as *Notch3*, *Epas1*, *Col18a1* and *Nr2f2*, suggesting a particular EC-modulating function not shared with other CAF subtypes. Using Nidogen-2 as a marker of this population, the authors observed vCAFs tightly associated with blood vessels in early stages of tumor development that were detached from vessels at later stages. These cells may originate from a pool of perivascular cells (i.e. mural cells) that over the course of tumor progression invade the tumor stroma. Accordingly, vCAFs express pericyte markers such as *Cspg4*, *Rgs5*, *Pdgfrb*, and *Des*, albeit at lower levels. Similarly, in human intrahepatic cholangiocarcinoma, a study identified CD146-positive CAFs as vCAFs exhibiting high expression of microvasculature signatures and IL6, and promoting cancer cell malignancy [36]. Notably, these CD146^+^ vCAFs were predominantly located in the tumor core, while other CAFs expressing POSTN were localized in the invasive tumor front. Additionally, other pathological fibroblasts can present a particular identity dictated by their localization within perivascular areas and interaction with ECs. For example, in rheumatoid arthritis, synovial fibroblasts upregulate NOTCH3 and Notch target genes due to signals from vascular ECs, significantly impacting inflammation and joint damage [105].

**Fig. 3:**
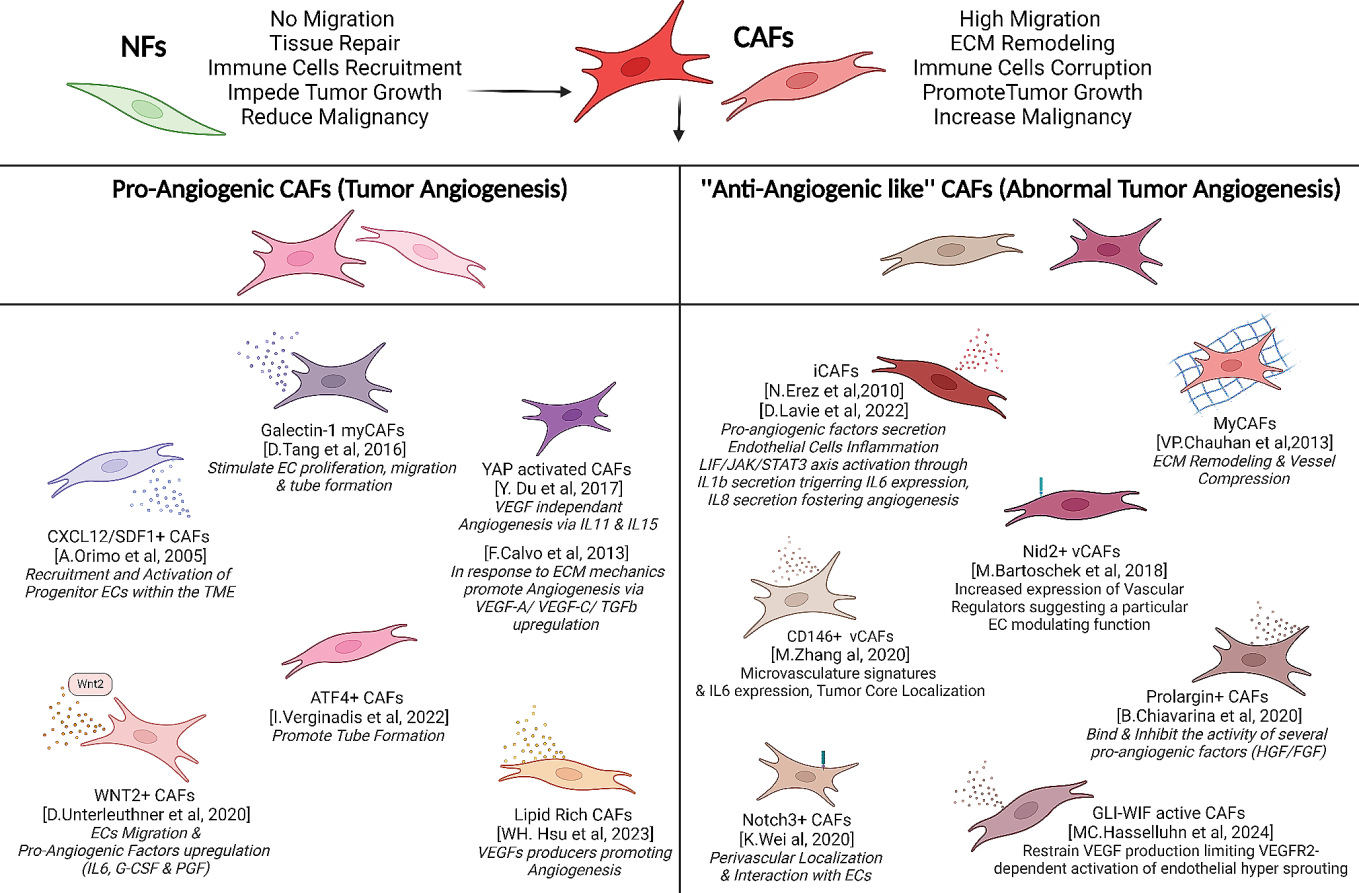
Identified CAFs implicated in tumor angiogenesis and the generation of abnormal tumor vasculature. Table summarizing the wide variety of CAFs implicated in pro- or anti- angiogenic processes. Due to the different origins of CAFs and the different subtypes, CAFs functions in tumor Angiogenesis can be ambivalent

Collectively, these studies suggest a level of specialization among CAFs concerning pro-angiogenic behavior or association with tumor vessels, necessitating further investigation. Understanding the specific contributions of the cell of origin to these phenotypes, particularly the relationship between vCAFs and perivascular cells, remains a key area of interest. Recent studies are beginning to uncover the distinct role of tumor-associated pericytes as an independent entity from CAFs [106]. Whether these distinct CAF phenotypes signify particular functions associated with vascular regulation and unique distributions within tumors or represent remnants of their lineage (e.g., mural cell- or EC-derived CAFs) is yet to be determined [107]. Future studies employing techniques like scRNAseq and spatial transcriptomics to map tissue/tumor cell composition and cell communication as well as lineage tracing analyses of CAF subtype cellular origins are expected to provide further evidence on the specific pro- or anti-angiogenic roles of CAF subpopulations (Fig. 3).

## Other mechanisms associated with tumor vasculature and interplay with CAFs

**Fig. 4:**
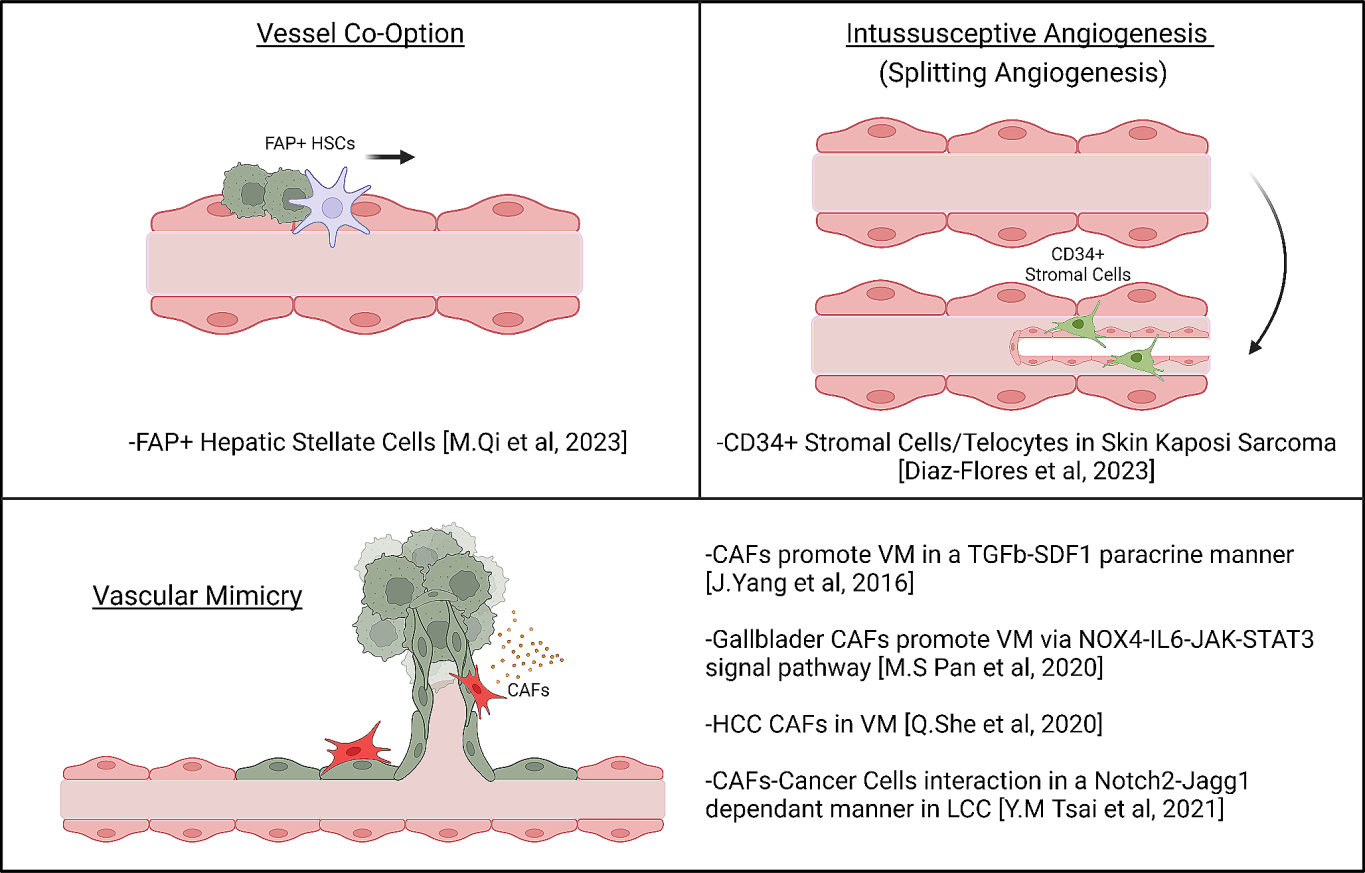
Evidence supporting a potential role of CAFs in alternative mechanisms of tumor neovascularization. Schematic representation of the potential implication of CAFs in processes associated with the formation of aberrant vasculature such as VM), Vessel Co-option or Intussusceptive Angiogenesis (Splitting Angiogenesis)

The evolution of tumor neovascularization involves diverse mechanisms beyond conventional angiogenesis, particularly in highly metastatic tumors or those resistant to anti-VEGF therapies. These alternative mechanisms secure a continuous blood supply for tumors and are not solely reliant on compensatory expression of alternative pro-angiogenic factors to overcome VEGF inhibition. Instead, they involve emergent behaviors and characteristics of ECs that significantly impact tumor progression. These mechanisms include various interactions between cancer cells and stromal cells, both through angiogenic (such as splitting angiogenesis) and non-angiogenic processes like vascular mimicry (VM) and vessel co-option. These observations underscore the complexity of tumor vascularization, emphasizing that the distinctive vascular state of a tumor is not solely dictated by its nutrient and oxygen demands. Rather, it reflects the intricate interactions among different components of the TME. Importantly, these processes might also be influenced or modulated by the actions of CAFs, suggesting an additional layer of CAF involvement in the regulation of tumor vasculature (Fig. 4).

### Vascular mimicry (VM)

VM presents a distinctive mode of tumor neovascularization, divergent from classical angiogenesis, as it allows tumors to establish a blood supply independently of ECs [108]. In VM, specialized cancer cells exhibit the ability to create microvessel-like structures or integrate into existing vessels, forming conduits lined by tumor cells expressing endothelial markers. These structures often possess anti-coagulant factors and can anastomose with the host vasculature. Alternatively, tumors may develop matrix-embedded vascular networks that facilitate the transport of plasma, blood cells, oxygen, and nutrients. Typically, these aberrant VM vessels are found in hypoxic and compressed regions of the tumor and tend to exhibit poor perfusion.

Regarding the involvement of CAFs in VM, emerging evidence suggests their potential modulatory role in this process. Initial studies revealed that conditioned media from CAFs prompted increased expression of genes associated with VM and facilitated the formation of VM structures in hepatocellular carcinoma cells [109]. Subsequent investigations provided deeper mechanistic insights into this phenomenon. In hepatocellular carcinoma, CAF-derived factors like TGFβ and SDF1 were found to promote VM formation both in vitro and in vivo, partly by inducing the expression of VE-Cadherin, MMP2, and LAMC2 in cancer cells [110].. Alternatively, Tsai et al. demonstrated that CAFs supported the formation capillary-like structures in cancer cells through the establishment of heterotypic Notch2-Jagged1 cell-cell contacts [111]. Notably, inhibition of Notch signaling in this context increased the therapeutic effectiveness of anti-VEGF treatments. Moreover, gallbladder CAFs have been identified to induce angiogenesis-related transcriptional states in gallbladder cancer cells, enhancing their VM potential and aggressiveness via NOX4 induction [112]. Here, CAF-derived IL6 triggered JAK/STAT3 activation in cancer cells, resulting in NOX4 upregulation.

### Vessel co-option

Vessel co-option represents a non-angiogenic mechanism in which cancer cells utilize pre-existing tissue blood vessels to sustain their growth, spread, and therapeutic resistance [113]. This process involves cancer cells migrating toward highly vascularized areas of the adjacent non-malignant tissue, exploiting these blood vessels for resources. As it operates independently of angiogenesis, vessel co-option has been implicated in conferring resistance to ATT. Although the precise molecular mechanisms driving vessel co-option remain elusive, processes affecting cancer cell adhesion and invasion are believed to be involved. Thus, factors within the TME influencing these cancer cell characteristics may impact vessel co-option. For instance, in AAT-resistant liver metastases, FAPα + hepatic stellate cells were found to be associated with vessel co-option [114]. Mechanistically, AAT induces the expression of FGFBP1 in resistant/surviving cancer cells. This factor promotes the recruitment and activation of FAPα^+^ stellate cells, triggering the secretion of CXCL5 and inducing epithelial-mesenchymal transition in cancer cells, facilitating their invasion and promoting vessel co-option. Notably, disrupting this specific mechanism reduced tumor progression and resistance to ATT, highlighting the significance of CAFs in the emergence of therapy-resistant tumors.

### Intussusception or splitting angiogenesis

Intussusception, also known as splitting angiogenesis, is a vascular remodeling process wherein a pre-existing blood vessel splits into two new vessels [115]. This mechanism facilitates the generation of new vascular branches, expanding the potential coverage area of the vasculature and enhancing nutrient and oxygen accessibility, albeit at the expense of reducing the average vessel size. The precise pathobiological relevance and regulatory mechanisms of intussusception are not yet fully characterized. However, intussusception is known to involve the activation of MT1-MMP, which cleaves Thrombospondin-1 to generate c-terminal fragments. These fragments then bind αvβ3 integrin leading to nitric oxide production followed by vasodilatation, pillar formation and division of the vessel into two distinct compartments, which then form two different vessels [116]. Regarding stromal regulation of intussusception, in Kaposi Sarcoma a CD34 + stromal cell population located at the external layer of the pre-existing blood vessels has been shown to participate in splitting angiogenesis and the formation of neo-vessel [117].

## TECs influence on CAF behavior

The influence of TECs on the behavior and emergence of CAFs is a relatively less explored aspect compared to the profound impact of CAFs on TECs and tumor vasculature. However, there are emerging perspectives on potential interactions between TECs and CAFs that warrant further investigation.

One potential influence may involve the direct contribution of ECs/TECs to the emergence of CAFs. The plasticity of ECs provides them with the potential to respond to specific signals and transdifferentiate into mesenchymal cells through a process called “endothelial-to-mesenchymal transition”(EndMT) [118]. This process is characterized by the loss of EC features and gain of mesenchymal traits, such as loss of cell-cell adhesions and delamination, changes in morphology and polarity, and increased migration. EndMT is a developmental process that can be triggered in adult tissues under pathological inflammation, giving rise to myofibroblasts and contributing to fibrosis. Since EndMT-derived cells present characteristics and functions associated with CAFs, ECs have been postulated as an additional source of CAFs in certain tumors [119]. Intriguingly, EndMT-derived cell characteristics may be reminiscent of certain CAF subpopulations identified through scRNAseq, and particularly of the previously mentioned vCAF subtype [35, 36]. Nevertheless, the exact origin of vCAFs remains controversial as they appear to emerge from mural cells undergoing pathological activation. Regardless of their actual cell of origin, it is increasingly evident that both EndMT-derived cells or EC-associated cells are contributing to what is referred as the CAF pool in tumors, but further investigation is required to ascertain the exact role of these CAF subtypes in tumorigenesis and their mechanisms of action.

Additionally, TECs may also influence CAFs as a significant source of signaling factors within the TME. Thus, it is likely that the expression of cytokine/chemokines by TECs will not only affect immune cell recruitment and cancer cell malignancy, but also alter the behavior of adjacent fibroblasts in tumors. TECs have been shown to express and secrete factors such as TGFβ and PDGF with a paramount importance in the recruitment of fibroblasts and its activation towards myCAF phenotypes. Similarly, TECs producing inflammatory signals like IL6 and CCL2 might contribute to the activation of iCAF programs. Although there is emerging evidence suggesting a connection between inflamed vasculature and fibrosis [73], data demonstrating how these processes precisely influence CAF behavior and impact tumor progression remains elusive and requires further investigation to establish a definitive link.

Noteworthy, many of the potential effects of abnormal tumor vasculature or activated TECs in CAFs may be a consequence of indirect interactions. For example, it is to be expected that the complex activity of TECs in modulating the immune cell milieu may have a downstream effect on the surrounding CAFs. This is an event that has been observed in fibrotic disorders triggered by EC injury such as scleroderma [73]. In this pathology, the microvasculature is the primary injury site and results in an imbalance of angiogenic and inflammatory processes in ECs. In such cases, ECs undergoing injury alter the expression of adhesion molecules and cytokines, which in turn affect the recruitment and activity of inflammatory cells, leading to myofibroblast transformation and fibrosis [120]. This sequence of events highlights the intricate relationship between ECs, inflammatory responses, and fibroblast activation in diseases associated with abnormal vasculature.

An additional indirect effect of abnormal vasculature in modulating CAF behavior is the impact of hypoxic environments in CAF activation, albeit this is still controverse. Thus, hypoxia has been shown to inactivate CAFs by downregulation of myCAF characteristics such as contractile force, ECM remodeling and promotion of cancer cell invasion in a HIF1α-dependent manner [121]. Conversely, hypoxia might also promote CAF activation and myofibroblast-like phenotypes by inducing the expression of TGFβ [122]. Alternatively, it has also been shown that hypoxia may also increase IL1α expression and therefore induce iCAF characteristics [123, 124]. The observed disparity in these findings might be attributed to tissue-specific or regional differences within tumors. It is also important to highlight that hypoxia may influence cells not only through well characterized signaling cascades, but also via less known metabolic cross-talks that may affect both TEC and CAFs.

Altogether, additional studies investigating the specific interaction between TECs and different CAF subsets are required to ascertain the influence of abnormal vasculature in modulating CAF emergence and composition in tumors. A fascinating possibility lies in the bidirectional influence exerted by ECs and stromal fibroblasts, and how this reciprocal signaling is altered in tumorigenesis. In normal physiology, blood vessels rely on bidirectional signaling involving ligand-receptor and receptor-receptor interactions between ECs and mural mesenchymal cells (such as pericytes) for stabilization [125]. Disruption of these interactions could potentially lead to detachment of mural cells and their subsequent activation, contributing to the pool of CAFs released into the TME [126].

In agreement, the concept of pericyte-derived CAFs is starting to emerge and there is a relative overlap between tumor pericyte markers and markers for distinct CAF subtypes [107]. Additionally, non-vascular fibroblasts or mesenchymal cells may be recruited and altered by signals emanating for TECs to promote their non-physiological interaction with the vascular bed, replacing bona fide mural cells and contributing to the chaotic tumor vasculature. In this scenario, the substitution of mural cells with alternative mesenchymal cells from the TME, which may lack regulatory mechanisms to control their cellular contractility or ECM production/remodeling activities, may also contribute to the aberrant phenotype of tumor vasculature. Thus, considering the heterotypic interactions CAFs have been shown to establish with cancer cells to facilitate collective invasion [23], it is plausible that similar interactions might exist between CAFs and TECs. Whether similar heterotypic interactions can be formed between CAFs and TEC is a possibility that warrants further research.

Thus, CAFs have been shown to generate heterotypic interactions with cancer cells to propel cancer cell collective invasion. Whether similar heterotypic interactions can be formed between CAFs and TEC is a possibility that warrants further research. CAFs express N-cadherin, which is also expressed by ECs and pericytes and is critical in the attachment of pericytes to the vascular wall [127], suggesting that CAFs may interact through N-cadherin with ECs. Alternatively, bidirectional modulation between ECs and CAFs may involve other juxtacrine mechanisms such as Notch signaling. Dysregulation of Notch signaling has been associated with vascular defects [61]. In other contexts, analyses of EC-fibroblast interaction in rheumatoid arthritis suggest the establishment of a signaling network involving Notch receptors between both cell types that promotes inflammation [105], which may also be relevant in cancer.

Overall, while the understanding of TEC-mediated modulation of CAF behavior is still in its early stages, emerging evidence suggests potential interactions between ECs and fibroblasts within the TME, shedding light on the complexity of TME regulation and its impact on tumor progression. Further studies are needed to delineate the exact mechanisms and functional consequences of these interactions in the context of cancer progression.

## Harnessing CAFs to normalize abnormal vasculature: opportunities and challenges

Althogether, it is evident that CAFs play a key role in the abnormal phenotype and architecture of tumor vasculature, and data is emerging in the complex bidirectional interaction between CAFs and TECs in modulating tumor promoting TMEs. These will probably have critical roles in tumor progression, dissemination, and responses to therapy. Understanding the interplay between CAFs and TECs offers potential avenues for improving anti-cancer therapeutics and stratifying patient treatments.

Notably, associations between reactive stroma and drug resistance are emerging, with stroma-associated gene signatures predicting responses to therapy [128]. It is becoming clear that CAFs can affect anticancer drug efficacy and are key players in promoting cancer cell evasion to therapies [20]. Mechanisms of resistance involving CAFs include the modulation of cancer cell–ECM interactions, cancer-CAF adhesion and cytokine- or chemokine-mediated signaling pathways. On general terms, these factors can promote pro-survival signaling or enable the reactivation/rewiring of critical signaling cascades that are targeted by therapeutics [4]. In addition, the modulation of CAFs or particular subsets of CAFs is showing potential to increase the efficacy of immunotherapy approaches in preclinical models by reducing the amount of immune suppressive phenotypes and increasing T cell recruitment and activity [21, 33].

In relation to the topic of this review, CAFs have also been shown to participate in AAT resistance through several mechanisms. The therapeutic action of ATTs is to prevent the excessive angiogenesis characteristic of most solid tumors to starve cancer cells and induce necrosis and tumor reduction. For that, different inhibitors targeting the main angiogenic pathway in ECs (i.e. VEGF) have been developed, such as bevacizumab, sunitinib, sorafenib, and pazopanib. This type of therapies presents certain limitations and off-targets effects that limited their clinical usage, but they have shown benefits in combination with chemotherapy regimes in some tumor types. However, responses are very heterogeneous due to the emergence of compensatory mechanisms leading to treatment refraction or acquired resistance. At the forefront of this mechanisms of resistance is the establishment of pro-angiogenic programs that sustain EC proliferation in the absence of VEGF signaling. These programs can be activated by cancer cells but also by CAFs. Thus, CAFs have shown to promote angiogenesis through several VEGF-independent mechanisms that suggest that they may have a key role in acquired resistance to AAT. For instance, CAFs from anti-VEGF-resistant tumors upregulate PDGF-C, supporting angiogenesis even in the absence of VEGF signaling. Targeting PDGF-C in combination with anti-VEGF treatments showed promising results in inhibiting angiogenesis and slowing tumor growth [129]. In addition, analyses in vitro using co-cultures of ovarian cancer patient-derived CAFs and HUVECs showed that CAFs presented a notorious pro-angiogenic effect that persisted during AAT, suggesting their impact on therapeutic efficacy [130]. These examples illustrate that the VEGF-independent pro-angiogenic factors produced by CAFs can affect the efficacy of AAT, that may be extendable to other factors still to be validated (i.e. interleukins, FGF). As previously discussed, CAFs have also been shown to participate in VM and vessel co-option, which are processes associated with AAT resistance. Noteworthy, AAT based in tyrosine receptor inhibitors of VEGFR like sunitinib in clear cell renal carcinoma has shown to be associated with a considerable increase in CAFs in patient specimens, which correlated with enhanced lymphangiogenesis, lymph node metastasis and shorter survival. This paradoxical effect may be associated with alternative mechanisms of resistance by promoting features of cancer aggressiveness, and also highlights additional limitations for this type of approaches in certain tumor types [131].

To overcome limitations associated to AATs, a novel approach to target the tumor vasculature is emerging and involves the restauration of aberrant tumor vasculature (i.e. vascular normalization). This strategy aims at limiting angiogenesis as well as restoring the normal function and architecture of tumor blood vessels enhancing their barrier function (increased EC-EC junctions, vascular basement membrane formation and attachment of mural cells) and reducing vessel compression (limit excessive ECM deposition and mechanical forces exerted over vessels) [132]. Importantly, this type of approaches will in principle overcome most of the main limitations of AATs and in particular will enable the correct supply of blood to the tumors, enhancing the penetration of combinatorial anti-cancer therapeutics (i.e. chemotherapy or immunotherapy) or immune cells to the tumor. However, decompression of tumor vessels is still relatively unexplored, as there is a current lack of physiologically relevant models to study this in detail, which has resulted in a poor understanding of the process. Nevertheless, reducing collagen and hyaluronic acid levels in preclinical models has shown promising results. Thus, enzymatic targeting of hyaluronan in pancreatic cancer models reduced solid stress and expanded the microvasculature, enhancing the effect of chemotherapy treatments [133, 134]. Furthermore, inhibiting collagen I synthesis improved the delivery of nanotherapeutics in tumors [135]. More recently, Chitty et al. showed that preventing excessive collagen crosslinking and stabilization through the use of a pan-lysyl oxidase inhibitor reduced tumor desmoplasia and stiffness in preclinical models of pancreatic cancer. Importantly, these effects were accompanied by an improved perfusion in tumors that enhanced the efficacy of chemotherapeutics [136]. The significant role of CAFs in promoting aberrant tumor vasculature and ECM deposition suggests their active participation in tumor vessel leakiness and impaired drug delivery [89]. In fact, Chitty et al. described through in vitro analyses that the primary cellular target of the pan-lysyl oxidase inhibitor were the CAFs [136], albeit their study did not investigate aberrant tumor vasculature. In a recent study, Chauhan et al. showed that administration of the angiotensin inhibitor losartan in preclinical models reduced CAF density and activation, leading to a significant decrease in collagen and hyaluronan production [137], which are directly associated with aberrant vasculature and poor perfusion [133, 134].

**Fig. 5:**
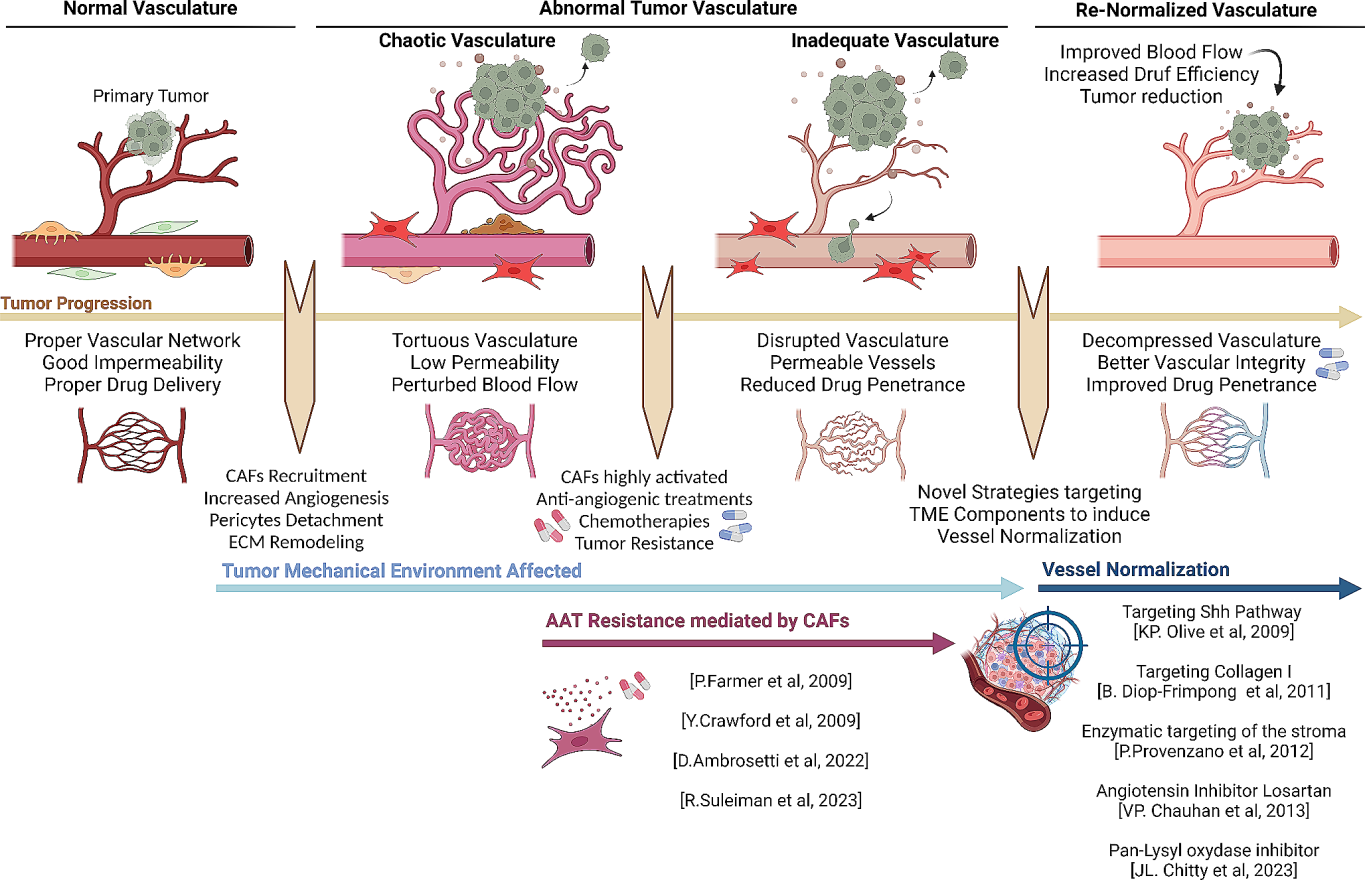
Generation of abnormal vasculature and persistence of aggressive and refractory TMEs. Diagram illustrating the generation of abnormal tumor vasculature, the persistence of aggressive and treatment-refractory TMEs, and the potential avenues for normalizing approaches. The specific role of CAFs in the development of abnormal tumor vasculature and resistance to AAT is also shown. Current evidence supporting strategies targeting CAF-related processes to alleviate vascular compression, enhance vascular integrity, facilitate improved blood flow, and enhance the penetration of anti-cancer drugs is highlighted

These findings underline the therapeutic implications of targeting the interactions between TECs and CAFs beyond anti-angiogenic strategies. Co-targeting CAFs in combination with established cancer therapeutics may mitigate CAF-dependent mechanisms of therapeutic resistance, offering new directions for improving treatment efficacy in cancer patients (Fig. 5). It is important to highlight that some of these perturbations are only effective in chemotherapeutic contexts (i.e. increase the delivery and efficacy of standard therapeutics) whilst promoting adverse effects in untreated tumors. It is therefore critical to acknowledge that altering TME homeostasis can unexpectedly lead to more aggressive tumors as well as providing therapeutic opportunities. An illustrative example is represented by the initial approach to relieve desmoplasia in PDAC by pharmaceutically and genetically blocking SHH signaling in CAFs, that counterintuitively resulted in increased tumor growth and more malignant tumors [138]. Interestingly, this particular treatment was associated with increased intratumoral vascular density, blood perfusion and drug delivery, improving chemotherapy outcomes in preclinical models. On the other hand, blocking angiogenesis can halt tumor growth but promote hypoxia and more malignant cancer phenotypes. Thus, there is a delicate balance between poorly vascularized tumors (hypoxia/malignancy/poor drug perfusion) vs. good perfused tumors (tumor growth/efficient drug delivery) that requires accurate assessment and fine tuning. As previously discussed, vascular normalization approaches may, at least in principle, overcome this problematic. However, there is a risk that these approaches are not profound enough and that certain tumor areas remain poorly irrigated and at risk of becoming highly aggressive. An alternative approach to circumvent homeostatic dysregulation associated with CAF elimination involves the modulation of specific CAF subsets or the inhibition of specific CAF-dependent processes in well-defined tumoral/therapeutic contexts. On the other hand, tumor resilience, adaptation and evolution are key aspects impacting in the emergence of therapeutic resistance to all types of anticancer treatments. Even though stromal cells are genetically stable and therefore less adapted to profound phenotypic changes, both fibroblasts/CAFs and EC/TECs present inherent levels of plasticity. It is therefore possible that unforeseen phenotypes and/or mechanisms may emerge during CAF- or TEC-directed treatments, leading to therapy evasion. For example, recent studies demonstrated differential activities associated to Col1-expressing myCAFs. In hepatocellular carcinoma, Col1-expressing myofibroblastic CAFs promoted tumor development through increased stiffness and TAZ activation in pretumoural hepatocytes, and through activation of discoidin domain receptor 1 in established tumors [31]. Intriguingly, in the same context, myCAF-expressed Col1 mechanically restrained tumor growth, overriding its own stiffness-induced signaling [29]. In PDAC, specific deletion of *Col1* in SMA-expressing myCAFs accelerated tumor progression by promoting SOX9 activation and Cxcl5 upregulation in cancer cells. This led to an alteration in immune composition in tumors, with recruitment of myeloid-derived suppressor cells and suppression of CD8^+^ T cells [28]. Thus, particular the cellular context being targeted needs to be taking into account, since similar strategies may lead to divergent outcomes when applied to different tumor types or at different stages of tumor development, and may sensitize tumors to certain therapies whilst reducing efficacy for others.

Finally, as with any approach targeting normal cells within tumors, careful examination of potential systemic effects altering organ function is paramount. Targeting CAF and/or TECs may also affect processes required in normal physiology (wound healing, tissue regeneration, vascular function, renal function, immune responses), so it is critical to identify tumor specific mechanisms as well as to carefully define safety levels of administration and contraindications.

## Concluding remarks and future directions

The intricate interplay between CAFs and TECs is increasingly recognized as pivotal in shaping the TME, profoundly impacting various facets of tumor progression and responses to treatment. While advancements have shed light on how CAFs influence TECs behavior, contributing to abnormal tumor vasculature and therapeutic resistance, there remains a significant gap in understanding how TECs reciprocally affect CAFs emergence and behavior. Further exploration is warranted to unveil how abnormal vasculature influences the emergence and roles of distinct CAF subsets within tumors or identifies specialized subpopulations particularly associated with this interplay. Exploring the direct crosstalk between these two stromal cell populations is crucial, understanding if they mutually sustain and impact each other within the tumor. Based on this insight, there is an opportunity to develop novel strategies aimed at disrupting this malignant communication occurring within the TME.

The substantial impact of CAFs within the TME underscores their influence on drug efficacy and cancer cell responses to therapies. Their pivotal role in fostering a supportive environment for tumor growth and evading treatments highlights their potential as valuable therapeutic targets. Strategies aimed at normalizing aberrant tumor vasculature have emerged as promising avenues to improve drug delivery and therapeutic effectiveness. Targeting CAFs directly through diverse approaches, such as modulating SHH signaling or inhibiting lysyl oxidase, shows promise in reshaping the TME and enhancing treatment outcomes. However, several challenges remain and usually revolve around our limited understanding of CAF/TEC specialization in terms of biological impact and role in cancer. Therefore, it is important to expand our current knowledge on the functional and molecular features of distinct subsets of CAF/TECs, which will probably be tissue or cancer specific. It is also critical to expand this characterization to certain contexts where stromal cell features are not well defined. In particular, stromal responses to anticancer therapies and the potential impact of distinct subtypes emerging during treatment remains obscure. These analyses will undoubtedly provide key insights on the role of CAF/TEC subsets in refractory tumors. These studies should be accompanied by the development of robust ex vivo/in vivo methodology to model and deconstruct the complex interactions between the different stromal components and cancer cells, and their evolution during tumor progression and therapy. These will ideally recapitulate more faithfully the complex cellular interactions and distinct cellular subtypes observed in native tissues, and will provide tools for mechanistic dissection and target identification. For example, a recent study leveraged on human and murine PDAC explant models to identify a multicellular signaling network involving cancer cells, CAFs and TECs that suppress angiogenesis in this type of cancer [78]. These improved models will also allow the tracking of stromal cell dynamics during treatment (including CAF- or TEC-directed therapies) to investigate CAF/TEC subtype evolution and the emergence of resistant phenotypes. In recent years, the identification and characterization of CAF subsets has advanced considerably, informing the development of pharmacological or genetic strategies for their specific manipulation. Defining subsets and ascribing distinct biological roles will be more challenging for TECs, giving the high level of specialization of ECs and the diverse structures that they form (capillaries, veins, arteries, lymphatics). But efforts should be made to improve our current models, where certain phenotypes (anergy and inflammatory phenotypes for TECs; immune modulatory features for CAFs) are extremely difficult to recapitulate. Altogether, these advances will enable the development of robust and standardized methods for detecting CAF/TECs in tissues, assessing their behavior in vitro and in vivo, and deconstruct their interactions. Ultimately, these may inform new therapeutic approaches and associated biomarkers to improve the percentage of patients that respond and expand their survival.

In summary, the intricate interrelationship between CAFs and TECs plays a pivotal role in shaping the TME, influencing therapy responses, and disease progression. Targeting CAFs in conjunction with conventional cancer treatments holds promise in mitigating CAF-mediated therapeutic resistance and improving treatment outcomes. Deciphering the complexities of CAF-TEC interactions holds substantial potential to uncover critical mechanisms underlying tumor vasculature and provide novel insights into tumor progression. This understanding could pave the way for innovative therapeutic strategies that target these multifaceted interactions, ultimately enhancing the efficacy of anticancer approaches.

## Data Availability

No datasets were generated or analysed during the current study.
